# High levels of variation in *Salix* lignocellulose genes revealed using poplar genomic resources

**DOI:** 10.1186/1754-6834-6-114

**Published:** 2013-08-07

**Authors:** Aude C Perdereau, Gerry C Douglas, Trevor R Hodkinson, Colin T Kelleher

**Affiliations:** 1 Teagasc, Agriculture and Food Development Authority, Kinsealy Research Centre, Malahide Road, Dublin, D17, Ireland; 2 Botany Building, School of Natural Sciences, Trinity College Dublin, Dublin, D2, Ireland; 3 DBN Plant Molecular Laboratory, National Botanic Gardens, Glasnevin, Dublin, D9, Ireland; 4 Trinity Centre for Biodiversity Research, Trinity College Dublin, Dublin, D2, Ireland

**Keywords:** Cellulose, Diversity, Lignin, *Populus*, *Salix*, SNPs, Cloning

## Abstract

**Background:**

Little is known about the levels of variation in lignin or other wood related genes in *Salix*, a genus that is being increasingly used for biomass and biofuel production. The lignin biosynthesis pathway is well characterized in a number of species, including the model tree *Populus*. We aimed to transfer the genomic resources already available in *Populus* to its sister genus *Salix* to assess levels of variation within genes involved in wood formation.

**Results:**

Amplification trials for 27 gene regions were undertaken in 40 *Salix* taxa. Twelve of these regions were sequenced. Alignment searches of the resulting sequences against reference databases, combined with phylogenetic analyses, showed the close similarity of these *Salix* sequences to *Populus*, confirming homology of the primer regions and indicating a high level of conservation within the wood formation genes. However, all sequences were found to vary considerably among *Salix* species, mainly as SNPs with a smaller number of insertions-deletions. Between 25 and 176 SNPs per kbp per gene region (in predicted exons) were discovered within *Salix*.

**Conclusions:**

The variation found is sizeable but not unexpected as it is based on interspecific and not intraspecific comparison; it is comparable to interspecific variation in *Populus*. The characterisation of genetic variation is a key process in pre-breeding and for the conservation and exploitation of genetic resources in *Salix*. This study characterises the variation in several lignocellulose gene markers for such purposes.

## Background

The Salicaceae family includes species important for biomass production in the *Salix* (willow) and *Populus* (poplar) genera. *Salix* comprises about 350–500 species [1] and is distributed over wide ecological and climatic zones ranging easterly from North America to China, excluding Australasia. *Salix* species show considerable variation in size, growth form and crown architecture, ranging from small dwarf shrubs in subgenus *Chamaetia*, middle-size shrubs in subgenus *Vetrix*, to the tree willows in subgenus *Salix*[2]. Demand for energy from renewable sources has provided a new impetus to growing willows in short-rotation coppice (SRC) plantations for bioenergy production because willow has the ability to grow fast without high maintenance or fertilizer input. Willow has the potential of producing energy that is greater than carbon neutral [3] and as such is seen as being a potential component of a green-house-gas reduction strategy [4].

Wood is one of the most important natural and renewable sources of energy and is therefore an important cost-effective alternative to burning fossil fuels. Lignin is one of its major components (25-35%) whose calorific value is similar to coal [5]. The evolution of lignin deposition is considered to be one of the key events during the evolution of primitive vascular plants [6,7]. Lignin has been one of the most intensively studied subjects in plant biochemistry for more than a century [8] and, consequently, the enzymes and processes of its biosynthesis pathway are well characterized [9]. Cellulose composes a greater proportion of plant cell walls than lignin, and is the world’s most abundant biopolymer [10]. The pathway leading to cellulose biosynthesis in higher plants is also well characterized. A number of studies have resulted in the publication of over 1,000 full-length or partial cellulose synthase (*CesA*) cDNAs or genes from representatives of many plants including cereals, fruits, or trees [11]. Little is known about sequence variation in the genes involved in these pathways especially in *Salix*. An understanding of sequence variation and potential genetic markers in these genes would be valuable for breeders and pre-breeders of *Salix* crops such as those for biomass and biofuel.

Willow’s closest relative, *Populus*, has a wealth of genomic resources including a complete genome sequence [12], physical and genetic maps [13,14], over 450,000 expressed sequence tags (ESTs) and thousands of full length cDNAs for a variety of genes. Assays have been developed to amplify poplar genes involved in disease resistance and wood formation [15-17]. A high degree of synteny between *Salix* and *Populus* genomes has been demonstrated, and the transfer of molecular markers between these genera has been achieved [18,19].

Specific genes in the lignin and cellulose biosynthesis pathways have been studied in poplar [15,20-22] but very little has been published on such candidate genes in *Salix*. A few studies have assessed the diversity of breeding collections and natural populations [23,24]. A number of studies have characterized genetic diversity for biomass willows [25] and developed markers for population genetic and breeding purposes [23]. Some have characterized ploidy in different species of *Salix*[26] and some have assessed species identity and classification [27]. These studies have tended to use either neutral or anonymous markers, as opposed to the approach in the current study, which involves targeting gene regions of known function. Little is known about genetic diversity within specific coding regions of the genome and especially in those regions responsible for wood formation. The monoploid chromosome number is 19, although many species are tetraploid and higher ploidy levels are also common [26,28].

In order to develop single nucleotide polymorphism (SNP) markers for future breeding efforts, we assessed 27 partial genes on a sample of 40 willow genotypes. We tested a broad range of species and varieties to enable greater scope to characterise variants across the genus. These samples include species from the three subgenera of *Salix*, nine commercial clones and six varieties from commercial basket makers. Genes assayed included those for lignin, cellulose and sucrose biosynthesis, growth hormone regulation, and transcription factors involved in lignin biosynthesis regulation. We tested the utility of *Populus* derived polymerase chain reaction (PCR) primers for amplification and sequencing of orthologous gene regions in *Salix* species. We aimed to quantify variation in these genes among *Salix* species. This is the first characterisation of nucleotide polymorphism from coding and non-coding regions for a set of lignocellulose genes in *Salix* species. These data can be used as an aid to decision making in selecting species and strains of willow for future breeding and growing. Polymorphisms and SNPs have potential to be used in selecting pre-breeding material and in association studies to link genotypic variants with specific phenotypes of interest.

## Results

### SNP discovery

After screening of the 27 loci using the primers designed from *Populus* species alignments [17], six loci showed one clear unique band following agarose gel electrophoresis in multiple samples (from 19 to 40 samples). The PCR conditions on seven other primers were changed by increasing the temperature and decreasing the time of the annealing step, to bring about a single band amplification. The amplicons were the same length as poplar in almost every case, from 411 to 1,204 bp. Further analysis was not carried out on the 14 genes that did not amplify successfully. Among them, nine failed to amplify and five showed multiple bands (See Additional file 1).

From this initial PCR (with very little optimisation), twelve loci that gave a strong single-band amplification were selected for sequencing in the *Salix* samples without cloning. These included genes in the lignin pathway (*4CL2*, *C3H1*, *C4H*, *HCT*, *PAL1*, *SAD*), genes for other wood components (*CesA1*, *CesA2* – cellulose synthase genes, *Kor1* - Korrigan), growth related genes (*GA*-*20*) and transcription factors (*Mybr2r3*, *Knat7*). However, out of these 12 loci, only eight yielded clear sequence traces (*C3H1*, *C4H*, *PAL1*, *SAD*, *CesA1*, *CesA2*, *Kor1*, *Knat7*). In order to further validate the results from the approach based on direct sequencing of PCR products from genomic DNA, PCR products from several loci were cloned. The regions cloned included amplicons of *4CL2*, *CAD*, *CesA1*, *GA*-*20*, *Knat7*, *Kor1*, *Mybr2r3* and *SAD* in a sample of genotypes including *Salix aurita*, *S*. *caprea*, *S*. *cinerea* ‘Tricolor’, *S*. *herbacea*, *S*. x*smithiana*, *S*. *viminalis*, the commercial clone ‘Tora’ and *Populus tremula*. In every case, transformed colonies were obtained successfully. A DNA insert was present in at least 50% of the cases. The products were sequenced as previously described.

BLAST (Basic Local Alignments Search Tool) searches against the NCBI GenBank database (http://blast.ncbi.nlm.nih.gov) and against Phytozome (http://www.phytozome.net) were performed on every sequence to test its identity. In all cases the target sequence was the top hit in the search results. BLAST results gave hits to several poplar species like *P*. *alba* L., *P*. *balsamifera* L., *P*. *deltoides* W. Bartram ex Marshall, *P*. *maximowiczii* Henry, *P*. *nigra* L., *P*. *tremula* L., *P*. *tremuloides* Michx., *P*. *trichocarpa* Torr. & A. Gray, and *P*. *tomentosa* Carrière.

**Figure 1:**
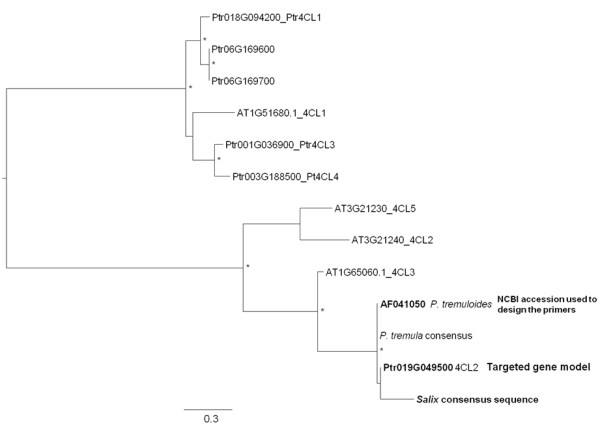
**Phylogenetic reconstruction of the genes used in this study.** Angiosperm gene family keyword search was performed into Phytozome (http://www.phytozome.net/search.php) and *A*. *thaliana* and *P*. *trichocarpa* genes were retrieved and aligned to a consensus *Salix* sequence generated from our study. Midpoint rooted, bootstrapped trees were generated using maximum likelihood analysis in PhylML 3.0 [29] with HKY85+G substitution model (estimated gamma shape parameter and four substitution rate categories) and NNI branch swapping. Bootstrapping included 1000 replicates with the same model and branch swapping. Asterisks indicate nodes with 80% or higher bootstrap support (Ptr: *P*. *trichocarpa*, AT: *A*. *thaliana*). The *4CL2* gene tree is given as an example (NCBI accession: AF041050, *Populus tremuloides* clone Pt4CL2 4-coumarate: CoA ligase mRNA, complete cds).

In order to test for possible paralogous amplification, further analyses were performed. For each gene, a phylogenetic reconstruction was performed (Figure 1). In every generated tree, the *Salix* consensus sequence was grouped with the targeted gene model with 80% or higher bootstrap support, thus indicating, in combination with the BLAST results that analogs and not paralogs were amplified.

From the 12 amplicons that were selected for sequencing and cloning, a maximum length of approximately 7,773 bp was sequenced per genotype. Between 19 and 28 genotypes were studied, for a total of 141,400 bp. The sequences were deposited in GenBank, under the accession numbers JQ345525-JQ345685 for the direct sequencing and KC491425-KC491628 for the cloned sequencing.

Multiple sequence alignments showed variability between genotypes; the variation consisted primarily of SNPs, but indels were also detected. Table 1 shows the number of SNPs per kbp among the *Salix* genotypes, among the *Populus* species and the total number of SNPs detected between the *Salix* samples and the *P*. *trichocarpa* gene model (http://www.phytozome.net/poplar). Exonic and intronic sequences were analysed relative to the latest version of *P*. *trichocarpa* genome (v3). Details of the number of SNPs per genotype compared to the *P*. *trichocarpa* are given in Additional file 2.

**Table 1:** **Variation for each gene region showing the*P***. ***trichocarpa*gene models used as reference**, **the number of*Salix*genotypes and*Populus*species studied**, **the total number of SNPs detected**, **the number of SNPs within*Salix*and within*Populus***; **the number of insertions**/**deletions**

**Gene region**	***P.trichocarpa*reference(Phytozome gene model)**^**a**^	**No.of*Salix*genotypes studied**	**No.of*Populus*species studied(See Table **2**)**	**Predicted Exons/introns**^**a**^	**Studied length (bp)**	**Total SNPs per kbp**^**b**^	**SNPs within*Salix*per kbp**^**c**^	**SNPs within*Populus*per kbp**^**c**^	**Total Indels**	**Total non synonymous sites among*Salix*per kbp**
A) DIRECT SEQUENCING										
*C3H1*	Potri.006G033300	23	5	Exon	347	118	63	40	0	6
				Intron	0	/	/	/	/	
*C4H*	Potri.013G157900	13	6	Exon	609	76	16	21	0	3
				Intron	0	/	/	/	/	
*CesA2*	Potri.005G194200	22	4	Exon	244	53	20	20	0	0
				Intron	87	195	92	11	2	
*Pal1*	Potri.006G126800	20	6	Exon	474	99	46	40	0	0
				Intron	0	/	/	/	/	
*CesA1*	Potri.002G257900	20	6	Exon	629	68	11	38	0	2
				Intron	130	208	146	31	3	
*Knat7*	Potri.001G112200	17	2	Exon	120	25	8	8	0	0
				Intron	95	284	221	42	3	
*Kor1*	Potri.003G151700	23	5	Exon	192	63	10	36	0	0
				Intron	117	179	111	17	0	
*SAD*	Potri.016G078300	15	4	Exon	395	142	78	30	1	28
				Intron	19	158	0	158	0	
B) COMBINED RESULTS (from direct sequencing and cloning)										
*CesA1*	Potri.002G257900	20	6	Exon	629	107	33	54	0	14
				Intron	130	315	177	46	8	
*Knat7*	Potri.001G112200	17	2	Exon	372	70	38	30	0	13
				Intron	392	209	117	56	15	
*Kor1*	Potri.003G151700	23	5	Exon	319	94	38	34	0	19
				Intron	438	201	98	23	11	
*SAD*	Potri.016G078300	15	4	Exon	582	165	93	24	1	38
				Intron	217	475	207	69	4	
C) CLONING										
*4CL2*	Potri.019G049500	7	2	Exon	249	116	48	8	0	20
				Intron	289	325	83	35	14	
*GA*-*20*	Potri.015G134600	6	2	Exon	620	176	58	105	0	27
				Intron	290	462	117	210	31	
*CAD*	Potri.009G095800	7	2	Exon	439	125	96	16	1	34
				Intron	436	273	128	76	21	
*Mybr2r3*	Potri.015G033600	6	2	Exon	351	117	23	26	1	11
				Intron	259	228	108	31	11	

^a^Based on *P*. *trichocarpa* v3 assembly retrieved from Phytozome (http://www.phytozome.net/poplar).

^b^(No. total SNPs / length) × 1,000.

^c^(No. SNPs within *Salix* or within *Populus* / length) × 1,000.

Both direct sequencing and cloning techniques were used.

The genomic regions investigated consisted of 72% coding and 28% non-coding sequences (as obtained from *P*. *trichocarpa* genome v3). Between 53 and 176 SNPs/kbp were detected in predicted exons per gene region (Table 1). Between 16 and 96 SNPs/kbp were discovered within *Salix* and between 8 and 105 SNPs/kbp within *Populus* (details of the accessions are given in Table 2). An average of 54 SNPs/kbp was found among commercial clones, 58 among hybrids and 57 among species. These differences were not significant under a *t*-test at a threshold of 5%. Between 195 and 475 SNPs/kbp were detected in predicted introns per gene region.

**Table 2:** ***Salix*species**, **hybrids and commercial clones and*Populus*accessions that were used in this study**

**Species/Clone**	**Subgenus**	**Source**	**Source ID**	**Poplar accessions**	**NCBI ID**
*S*. *alba* L. var. vitellina L. Stokes	Salix	NBG	1977.1871	*P*. *alba* x *P*. *grandidentata*	EU391631 (C3H1)
*S*. *aurita* L.	Vetrix	NBG	1988.0222	*P*. *alba* x *P*. *grandidentata*	GU324115 (Kor1)
*S*. *babylonica* L. var. pekinensis ‘Tortuosa’	Salix	NBG	1978.0029	*P*. *balsamifera* x *P*. *deltoides*	AJ438351 (C3H1)
*S*. *caprea* L.	Vetrix	NBG	1979.0307	*P*. *kitakamiensis*	D82815 (C4H)
*S*. *cinerea* L. ‘Tricolor’	Vetrix	NBG	2002.2187	*P*. *sieboldii* x *P*. *grandidentata*	D30656 (Pal1)
*S*. *elaeagnos* Scop. (= *S*. *incana* Schrank)	Vetrix	NBG	2001.2367	*P*. *tomentosa*	EU760387 (C4H)
*S*. *exigua* L.	Salix	NBG	2003.0719	*P*. *tomentosa*	FJ534554 (CesA1)
*S*. *fragilis* L.	Salix	NBG	1977.0902	*P*. *tomentosa*	HQ585873 (CesA2)
*S*. *glabra* Scop.	Vetrix	NBG	XX.004178	*P*. *tomentosa*	EU760386 (Pal1)
*S*. *gracilistyla* Miq. var. melanostachys	Vetrix	NBG	1976.0659	*P*. *tremula*	EU753093 (C3H1)
*S*. *herbacea* L.	Chamaetia	Wild collected, Ireland		*P*. *tremula* x *P*. *tremuloides*	AY573571 (CesA1)
*S*. *lucida* Muhlenb.	Salix	NBG	1986.0050	*P*. *tremula* x *P*. *tremuloides*	AY573572 (CesA2)
*S*. *pentandra* L.	Salix	NBG	1926.004139	*P*. *tremula* x *P*. *tremuloides*	AY660967 (Kor1)
*S*. *phylicifolia* L.	Vetrix	NBG	1970.0402	*P*. *tremula* x *P*. *tremuloides*	AY850131 (SAD)
*S*. *phylicifolia* L.	Vetrix	NBG	2008.1233	*P*. *tremuloides*	DQ522295 (C4H)
*S*. *rehderiana* Schneid.	Vetrix	NBG	XX.004165	*P*. *tremuloides*	AF527387 (CesA1)
*S*. *scouleriana* Barr.	Vetrix	NBG	1984.0752	*P*. *tremuloides*	AY535003 (Kor1)
*S*. *viminalis* L.	Vetrix	NBG	2004.0293	*P*. *tremuloides*	AF480619 (Pal1)
*S*. x*chrysocoma* Dode (= *S*. *alba* L. var. vitellina L. Stokes x*S*. *babylonica* L.)		NBG	XX.004181	*P*. *tremuloides*	AF273256 (SAD)
*S*. x*erdingeri* Kern (= *S*. *daphnoides* Vill. x*S*. *caprea* L.)		NBG	1925.004149	*P*. *tricho*x*P*. *deltoides*	AF302495 (C4H)
*S*. x*erythrofle*x*uosa* Rag. (= *S*. x*chrysocoma*x*S*. *matsudana*)		NBG	1986.0041	*P*. x*canadensis*	AM922197 (Pal1)
*S*. x*laurina* Sm. (= *S*. *caprea* L. x*S*. *phylicipholia*)		NBG	1977.1877	*P*. x*canescens* (*P*.*tremula*x*P*.*alba*)	AF081534 (CesA1)
*S*. x*rubens* ‘Basfordiana’ (= *S*. *alba* L. var. vitellina L. Stokes x*S*. *fragilis* L.)		NBG	XX.004137		
*S*. x*rubens* ‘Schrank’ (= *S*. *alba* L. x*S*. *fragilis* L.)		NBG	XX.004133		
*S*. x*rubens* Schrank ‘Basfordiana’ var. sanguinea		NBG	1981.1773		
*S*. x*rubra* Huds (*S*. *purpurea*x*S*. *viminalis* L.)		NBG	1981.1520		
*S*. x*smithiana* Willd. (= *S*. *cinerea* L x*S*. *viminalis* L.)		NBG	2004.0292		
*S*. *purpurea* L.	Vetrix	Joe Hogan	Joe Hogan1		
*S*. *viminalis* L.	Vetrix	Joe Hogan	Joe Hogan2		
*S*. *viminalis* L.	Vetrix	Joe Hogan	Joe Hogan3		
*S*. *purpurea* L.	Vetrix	Joe Hogan	Joe Hogan4		
‘Sven’: *S*. *viminalis L*. x (*S*. *schwerinii*x*S*. *viminalis*)		Svalöf Weibull (Sweden)			
‘Inger’: *S*. *triandra*x*S*. *viminalis*		Svalöf Weibull			
‘Tordis’: (*S*. *schwerinii*x*S*. *viminalis*) x*S*. *viminalis*		Svalöf Weibull			
‘Endurance’					
‘Tora’: *S*. *schwerinii*x*S*. *viminalis*		Svalöf Weibull			
‘Resolution’: (*S*. *viminalis*x (*S*. *schwerinii*x*S*. *viminalis*)) x (*S*. *viminalis*x (*S*. *viminalis*x*S*. *schwerinii*))		UK (European)			
‘Doris’: *S*. *dasyclados*		Sweden			
‘Terra Nova’: (*S triandra*x*S*. *viminalis*) x*S linderstipularis*		UK (European)			
‘Torhild’: (*S*. *schwerinii*x*S*. *viminalis*) x*S*. *viminalis*		Svalöf Weibull			
*P*. *tremula* L.		Wild collected, Ireland			

The subgenus, the source and the identification of each sample is shown. NBG=National Botanic Garden, Dublin. Joe Hogan = Commercial basket maker. NCBI = http://www.ncbi.nlm.nih.gov/.

The most frequent types of polymorphisms were C-T transitions for coding SNPs (40.5% of SNPs) and A-T transversions for non-coding SNPs (27.3%). The least frequent types were A-C and G-T transversions for coding SNPs (7.5%) and C-G transversions for non-coding SNPs (data not shown).

Between 0 and 22 non-synonymous SNPs were discovered among *Salix* genotypes per gene region, corresponding to between 0 and 38 SNPs/kbp (Table 1). Multiple indels were also identified. Among them, only three were located in coding regions. Two were multi-base indels (3 and 8 bp). One shift of the reading frame was detected in *SAD* for *S*. *herbacea* due to an 8 bp insertion at the homozygous state. This was confirmed by cloning where the insertion was detected in every haplotype of *S*. *herbacea*. A 3 bp insertion was detected in *Mybr2r3* for all *Salix* individuals compared to the reference *P*. *trichocarpa* and *P*. *tremula* sequences. A deletion of one bp was also detected in *CAD* for one haplotype of *Salix* ‘Tora’.

All the SNPs of *CesA1*, *Knat7*, *Kor1*, and *SAD* found from direct sequencing of amplicons from genomic DNA were confirmed by analysis of the sequences from the cloned amplicons. In addition, more SNPs were found in the cloned compared to the uncloned sequences (see Table 1B). This was as a result of an increase in the length of the sequences obtained from cloned PCR products and the fact that haplotypes could be resolved. When the same length is studied, 38 more SNPs were discovered in *CesA1*, 5 for *Knat7*, 13 for *Kor1*, and 15 for *SAD*. Between 400–500 more bp were sequenced via the cloning approach except for *CesA1* where the same length was observed. Therefore, 78 more SNPs in total were discovered for *Knat7*, 85 for *Kor1*, and 140 for *SAD* (data not shown). Cloning also allowed a better resolution of the indels, as between 4 and 12 more indels were found (all located in predicted non-coding regions).

A different sequence length was obtained for all cloned PCR products of *S*. *viminalis*, *S*. x*smithiana* and for one sequence of *Salix* ‘Tora’ for the SAD gene region. Indeed, sequences of c. 1,030 bp were obtained for these sequences compared to c. 800 bp for the other cloned individuals. Thus an additional 230 bp was obtained towards the end of the sequence after the last predicted intron and this was very different from the reference sequence.

Many indels were found in *4CL2*, *CAD*, *GA*-*20* and *Mybr2r3*, and this was the main reason why the sequences were not analysable without the cloning step (Table 1C). For *GA*-*20*, indels at a heterozygous level were found in nearly all the individuals analysed. Between 116 and 176 SNPs/kbp were found in total in predicted exons, and between 23 and 96 SNPs/kbp found within *Salix*. Between 228 and 436 SNPs/kbp in total were found in predicted introns (Table 1C).

Haplotype number was calculated after the cloning step using Arlequin 3.5. Between 1 and 8 haplotypes were found per individual (Table 3). *Salix cinerea* ‘Tricolor’ and *S*. x*smithiana* are polyploids (4x and 3x, respectively). The other genotypes are diploid (Table 3). However, excluding the 2 polyploid genotypes more than 2 haplotypes were found in 70% of the cases.

**Table 3:** **Number of haplotypes found in*Salix***

	**Ploidy**^**1**^	***CesA1***		***GA-20***		***Knat7***		***4CL2***		***Kor1***		***CAD***		***SAD***		***Mybr2r3***	
		**S**	**H**	**S**	**H**	**S**	**H**	**S**	**H**	**S**	**H**	**S**	**H**	**S**	**H**	**S**	**H**
*S*. x*smithiana*	3X	4	3	4	3	7	5	4	4	4	4	3	3	4	4	4	3
*S*. *caprea*	2X					3	1	4	2	4	3	5	5	4	3	4	2
*S*. *viminalis*	2X	4	3	4	3	4	4	4	2	4	4	5	5	4	4	4	3
*S*. *cinerea* ‘Tricolor’	4X			4	2	7	4	4	4	4	4	5	5	4	3	4	4
*S*. *aurita*	2X	4	3	2	2	8	1	4	2	4	2	7	7	4	3	4	4
*S*. *herbacea*	2X	4	4	4	3	8	8	4	3	4	4	5	4	3	3		
*S*. ‘Tora’	2X	4	2	3	2	8	4	4	2	4	4	7	7	5	5	4	3

^1^based from Thibault, 1998 [26] Stamati et al., 2003 [30] and MacAlpine et al., 2008 [31].

(S=number of sequences analysed, H=number of haplotypes found).

**Figure 2:**
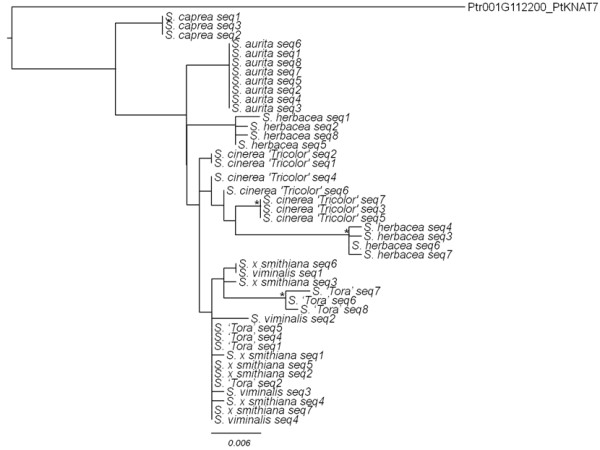
**Midpoint rooted, maximum likelood tree of *Knat7 *haplotypes with bootstrap values generated in PhyML with HKY85+G substitution model.** Asterisks indicate branches with 80% or higher bootstrap support. *Knat7* haplotype tree is given here as an example. Ptr001G112200_PtKNAT7: *P*. *trichocarpa*; similar to homeodomain transcription factor (KNAT7).

In order to further investigate this issue, midpoint rooted, bootstrapped maximum likelihood trees were generated in PhyML for each of the genes. The *Knat7* haplotype tree is given as an example (Figure 2). The tree grouped together the different haplotypes of a species in no more groups than the expected ploidy (although these groups were not strongly supported). There was very little difference between the haplotypes. The same pattern was found for the other genes (data not shown).

**Figure 3:**
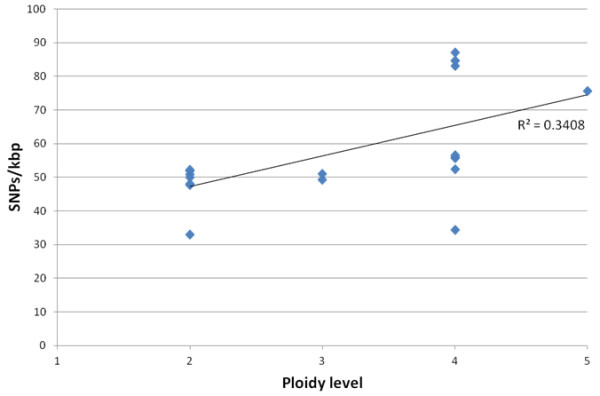
**Regression analysis of the putative ploidy level against the number of SNPs/kbp.** The trendline and R^2^ are shown.

Regression analysis was used to explore the relationship between DNA ploidy and number of SNPs/kbp on 20 *Salix* genotypes. It showed that there was a positive correlation between ploidy and the number of scored fragments (F_20_=8.94, P<0.005, R^2^=0.34) (Figure 3).

### Phylogenetic trees

**Figure 4:**
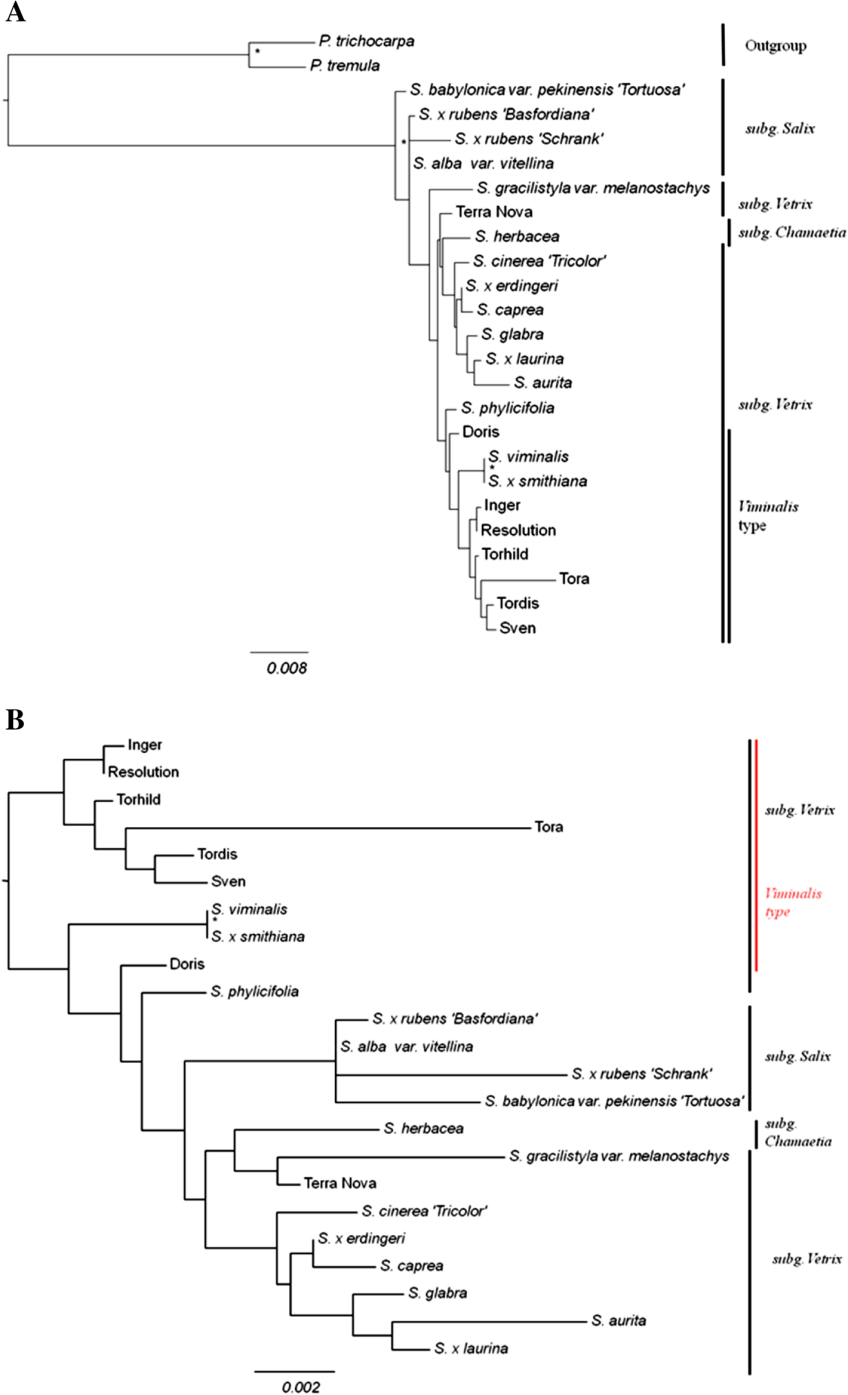
**Midpoint rooted**, **bootstrapped trees based on the eight genes analysed by direct sequencing (and the genotypes where most of the sequences were present)generated using maximum likelihood in PhyML with HKY85+G substitution model.** Asterisks indicate nodes with 50% or higher bootstrap support. The three *Salix* subgenera (according to most authorities) are indicated. Trees are shown with the *P*. *trichocarpa* gene model and *P*. *tremula* sequences included (Figure 4**A**) or excluded (Figure 4**B**).

In order to explore similarities or dissimilarities in the data, phylogenetic trees were generated with maximum parsimony in PAUP 4.0 and maximum likelihood in PhyML with HKY85+G. The two types of analysis gave the same results. Therefore only the trees from the PhyML analyses are shown (Figure 4). These trees are based on the eight genes analysed by direct sequencing and the genotypes where most of the sequences were present, with the *P*. *trichocarpa* gene model and *P*. *tremula* sequences included (Figure 4A) or excluded to improve resolution around the *Salix* samples (Figure 4B).

The trees give a visual indication of the genetic variability in the samples analysed. Although fully resolved, the trees are not strongly supported. However, the two poplars are separated from the *Salix* and the 3 subgenera are recognizable. The four *Salix* subg. *Salix* genotypes are grouped together, as well as most of the *Salix* subg. *Vetrix* genotypes. All the *viminalis* type genotypes are grouped together, *S*. *viminalis*, the commercial clones, and a hybrid *S*. *cinerea*x*S*. *viminalis*. *Salix herbacea* appears to be not so different from the subg. *Vetrix* genotypes.

## Discussion

We tested the transferability of *Populus* genomic resources (a set of PCR primers for multiple lignocellulose genes) to its sister genus *Salix* and obtained data on the level of sequence variation between and within the genera. Without much PCR optimisation we found that approximately 30% of the markers developed for *Populus tremuloides* worked in a broad sample of *Salix* genotypes. This is based on an approach of direct sequencing of PCR products from genomic DNA amplification in a variety of regions in a large array of genotypes. The cloning step on a set of *Salix* genotypes was added to validate the direct sequencing approach and to improve resolution of the data. This method was successful on all the genotypes and regions of the genome assayed. It increased the proportion of successful PCRs from 30 to 44%. This means that we can use almost one half of the primers designed for *Populus* on *Salix*. This level of transferability will prove very useful for the studies of natural populations of willows and for plant breeding, where marker availability is much lower than in poplar. The relatively low 30% success rate was partially due to the fact that we tested across such an array of species and genes and it contrasts with other studies that showed greater transferability [18]. It is likely that this rate could be increased with a more limited subset of targets and more optimisation, but for the purposes of this study we decided to focus on the regions that amplified in a majority of species without optimisation.

SNP discovery and transferability of *Populus* primers to *Salix* have been studied [18,19,32,33] but these studies were limited to a small number of species, in particular *S*. *viminalis*, *S*. *schwerinii*, and their hybrids. Studies on multiple *Salix* species have been done based on AFLP markers or a limited number of sequences from the chloroplast and nuclear ribosomal regions [34-37]. None have so far assessed variation in lignocellulose genes among *Salix* species.

Kelleher et al. [17] showed the same primers amplified successfully across a variety of *Populus* species. Here we show a subset also amplifies putative orthologs in *Salix*. A high level of gene synteny has already been shown between *Populus* and *Salix* based on high-density genetic maps [18,19] and the current data confirms the homology of amplicons between these genera in wood and cell wall related genes. In the current study, between 76 and 267 SNPs/kbp were discovered in a set of 12 genes. The number of SNPs detected is relatively high compared to other studies [17,38,39], partially because comparisons were made between genera and genotypes and not among individuals of the same species. These SNPs are a potential toolkit for future pre-breeding and will be tested for species specificity. When these data were compared to *Populus* accessions retrieved from NCBI, similar levels of variation were found among *Salix* and *Populus* species. Within *Salix*, the total variation among commercial clones and hybrids was lower than among species (68 SNPs and 42 SNPs vs 89 SNPs respectively, data not shown), but was not significant under a *t*-test at a threshold of 5%. These results are consistent with the fact that a narrow genetic pool is often used for breeding willows. For example, the *Salix* cultivars ‘Torhild’ and ‘Tordis’ are half-sibs with ‘Tora’ as the common female parent. Also the female parent SW930812 of ‘Resolution’ is a sibling of ‘Sven’. The majority of SRC willows currently grown in Europe are interspecific hybrids with some *Salix viminalis* in the pedigree. This species is favoured for SRC because it grows fast, coppices well and maintains a good growth form [40]. We show that there is a wealth of genetic variation within *Salix* that is as yet untapped.

Another issue to address is the possible polyploidy of some of the *Salix* genotypes studied. Suda [41] reported that *Salix* is one of the few woody genera with a large number of polyploid taxa. A study by Thibault [26] investigated polyploidy in 16 species of *Salix* including *S*. *alba* L. var. vitellina L. Stokes, *S*. *fragilis* L., *S*. *cinerea* ‘Tricolor’ and the hybrids *S*. x*rubens* Schrank and *S*. x*chrysocoma* Dode which were shown to be tetraploid. *Salix babylonica* var. *pekinensis* ‘Tortuosa’ has also been shown to be tetraploid [28]. The positive correlation we found between the number of scored markers and ploidy was consistent with results reported in other studies [36].

**Figure 5:**
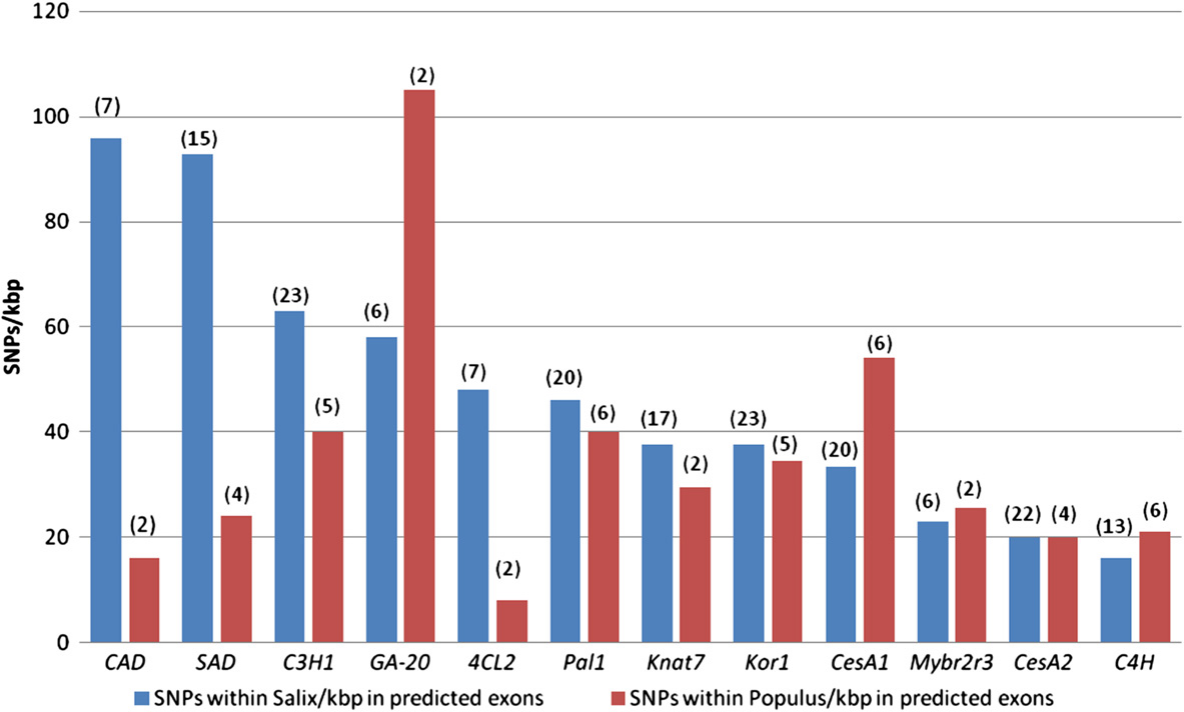
**Differences in the levels of SNPs in predicted exons within the genes found in *Salix *and *Populus.*** The data are ranked based on the levels of variation in *Salix*. The number of *Salix* genotypes or *Populus* species studied is indicated above each column.

When levels of variation are compared between *Salix* and *Populus* in predicted exons, half of the genes show a trend towards larger diversity in *Salix* compared to *Populus* (*Pal1*, *Knat7*, *Kor1*, *Mybr2r3*, *CesA2*, *C4H*) (Figure 5) and the remaining half show the opposite - *Populus* more variable than *Salix*. For example, the *CAD* gene is the most variable region in *Salix* but is one of the least variable in both the species of *Populus* studied. This could potentially be due to copy-number variation or differential regulation of wood formation in the 2 different genera, but further study is necessary to establish any patterns. Variation was found to be much lower in intraspecific studies of natural populations of *P*. *tremula*[38,39], where 17 SNPs/kbp were detected within five nuclear genes [38], 19 SNPs/kbp with 77 gene fragments [39] and 18 SNPs/kbp in *P*. *tremuloides*[17]. However, as the previous studies [17,38,39] are based on intraspecific variation it is understandable that the levels from the current study are much higher - 53 SNPs/kbp in *Salix* and 30 in *Populus* in the current study.

From the phylogenetic tree results (Figure 4), differentiation is shown among *Salix* species. Members of the different subgenera are often grouped together (although not strongly supported). All the commercial clones group close to *S*. *viminalis*, which is the main species used for willow breeding and thus the clones share sequence similarity with this.

This study also emphasizes that wood formation genes and more particularly the ones involved in the phenylpropanoid metabolism (*PAL1*, *C3H1*, *C4H*, *SAD*, *CAD*, *4CL2*) are conserved among two genera in Salicaceae. These results are consistent with a study by Hamberger et al. [42] who discovered considerable conservation among poplar, *Arabidopsis*, and rice genomes for nine phenylpropanoid gene families. While the genes show a high level of conservation, the difference in the mutation rates as shown in Figure 5 is of interest to understanding the development and evolution of wood formation genes within Salicaceae.

*PAL1*, *C3H1*, *C4H* and *4CL2* genes have relatively few non-synonymous sites (0, 6, 3 and 20 non-synonymous SNPs/kbp, respectively). These genes are upstream of the lignin pathway [9,42]. On the other hand, the *CAD* and *SAD* genes have more non-synonymous sites (34 and 38 non-synonymous SNPs/kbp, respectively). CAD enzyme is employed at the end of the monolignol biosynthetic pathway to convert the esters into their corresponding alcohols [43]. According to Li et al. [44], *SAD* is essential for the biosynthesis of syringyl monolignol and also intervenes at the end of lignin biosynthesis. Our results are consistent with the prediction that selective constraint is progressively relaxed along metabolic pathways [45]. Upstream genes are more pleiotropic, being required for a wider range of end products and thus tend to be more selectively constrained. However, another study on tobacco (*Nicotiana tabacum* L.) from Barakate et al. [46] has shown that *SAD* might not be involved in the last step in syringyl monolignol biosynthesis in wood-producing angiosperms. They have shown the existence of *SAD* in lignifying tissues but are not clear of its function. Moreover, it has also been hypothesized that SAD might be a dehydrogenase that directs monolignol precursors toward plant defense [47], which could explain the relaxed selective constraint found in this study.

With the cloning step, more regions have been sequenced and analysed on a subset of samples. It has been shown that orthologs have been amplified with confidence. More SNPs have been discovered than with direct sequencing from non-cloned amplicons. Results for haplotype number determination, by mere counting in Arlequin, gave more than 2 haplotypes in 70% of the cases for assumed diploid species. The different haplotypes found in each individual were similar except for a few SNPs as emphasized by the haplotype trees. BLAST searches and gene trees revealed that the reference used for the primer design and the targeted *Populus* gene model were closely related to the *Salix* sequences so it is most likely that these different haplotypes are not duplicates of another gene family member. Another possible explanation for excess haplotype number is that cloning emphasizes single-base substitution errors during the initial PCR. They are estimated to occur at a rate of 1–7 × 10^–4^ per base pair per cycle [48]. Some variants were possibly generated by enzyme slippage during sequencing especially for homopolymeric regions. It is also possible that some of the taxa, presumed to be diploid, are in fact polyploid.

## Conclusion

We have characterised a large number of new SNP markers for an array of willow species (*Salix* spp.), in 12 gene regions involved in the lignin and cellulose biosynthesis pathways. The poplar genome sequence and other poplar based resources were used as a reference and the resources were transferred to *Salix*. This is the first study to quantify levels of nucleotide polymorphism in a wide range of willows for such genes from the three subgenera of *Salix*. The results show that *Salix* harbours significant amounts of genetic variation at these loci (both between the different *Salix* species and between *Salix* and *Populus*). The study shows that the markers can be transferred from *Populus* to *Salix* in approximately half of the cases and justifies this approach for marker application and development in *Salix*. The data should be of interest and potential use to future work on pre-breeding programmes or association genetics in *Salix*.

## Methods

### Sample collection and DNA extraction

Leaf tissue samples from *Salix alba* L. var. *vitellina* L. Stokes, *S*. *aurita* L., *S*. *babylonica* L. var. *pekinensis* ‘*Tortuosa*’, *S*. *caprea* L., *S*. *cinerea* L. ‘*Tricolor*’, *S*. *elaeagnos* Scop., *S*. *exigua* L., *S*. *fragilis* L., *S*. *herbacea* L., *S*. *glabra* Scop., *S*. *gracilistyla* Miq. f. *melanostachys* (Makino) H.Ohashi, *S*. *lucida* Muhlenb., *S*. *pentandra* L., *S*. *phylicifolia* L., *S*. *rehderiana* Schneid., *S*. *scouleriana* Barr., and *S*. *viminalis* L. were obtained from living specimens at the National Botanic Gardens, Dublin, Ireland. Nine hybrids were also sampled from the Gardens. Samples of commercial clones such as ‘Sven’, ‘Inger’, ‘Tordis’, ‘Tora’, ‘Resolution’, ‘Doris’, ‘Terra Nova’, ‘Torhild’ and ‘Endurance’ were obtained from Teagasc Kinsealy Research Centre, Dublin, Ireland. Four varieties (mainly *S*. *viminalis* and *S*. *purpurea*) from an Irish basket maker in Galway completed the list (Table 2). A sample of *Populus tremula* L. collected from a wild population was also used as a template for the amplifications and sequencing.

Leaves were dehydrated and kept in plastic bags using silica gel. The tissue was disrupted using the Qiagen Tissuelyser II, and DNA was extracted using the DNeasy Plant Mini Kit by Qiagen Ltd. The DNA samples were kept frozen at −20°C. The resulting DNA was viewed and assessed for quality and quantity using agarose gel electrophoresis, ethidium bromide staining and a UV light.

### Gene regions and primer description

The gene regions tested included those of the lignin biosynthesis and regulation pathways, genes involved in the synthesis of other cell wall compounds such as cellulose, sucrose and korrigan (an endo-glucanase that is essential for proper cell wall formation) and one growth hormone (Table 4). A total of 27 gene fragments were studied. Fourteen gene regions are involved in the lignin biosynthesis, eight in lignin regulation as transcription factors, three in cellulose biosynthesis, one in sucrose biosynthesis and one in growth hormone regulation. The primers for these regions were developed from multiple species EST (Expressed Sequence Tag) alignments from *P*. *tremuloides* and *P*. *trichocarpa*[17].

**Table 4:** Gene regions assessed and their biochemical role

**Locus**	**Phytozome gene ID targeted**^**1**^	**Role**
*4CL1prom*	Not used	Lignin biosynthesis
*4CL1*	Not used	Lignin biosynthesis
*4CL2*	Potri.019G049500	Lignin biosynthesis
*AP2*	Not used	Putative lignin regulation
*BHLH144*	Not used	Putative lignin regulation
*BZIP47*	Not used	Putative lignin regulation
*BZIP9*	Not used	Putative lignin regulation
*C3H1*	Potri.006G033300	Lignin biosynthesis
*C4H*	Potri.013G157900	Lignin biosynthesis
*CAD*	Potri.009G095800	Lignin biosynthesis
*CCoAOMT*	Not used	Lignin biosynthesis
*CCR*	Not used	Lignin biosynthesis
*CesA1*	Potri.002G257900	Cellulose biosynthesis
*CesA2*	Potri.005G194200	Cellulose biosynthesis
*F5H*	Not used	Lignin biosynthesis
*GA*-*20*	Potri.015G134600	Growth hormone
*HCT*	Not used	Lignin biosynthesis
*Knat7*	Potri.001G112200	Putative lignin regulation
*Kor1*	Potri.003G151700	Korrigan – endoglucanase cell wall formation
*myb*	Not used	Transcription
*Myb63*	Not used	Putative lignin regulation
*Mybr2r3*	Potri.015G033600	Transcription
*PAL1*	Potri.006G126800	Lignin biosynthesis
*PAL2*	Not used	Lignin biosynthesis
*PTOMT1*	Not used	Lignin biosynthesis
*SAD*	Potri.016G078300	Lignin biosynthesis
*Sucrose synthase*	Not used	Sucrose biosynthesis

^1^Based on *P*. *trichocarpa* v3 from Phytozome (http://www.phytozome.net/poplar).

Details as in Kelleher et al. [17].

### Amplification of target genes

Primers from Kelleher et al. [17] were used for the amplification of 27 different gene regions of the nuclear genome. Primers were synthesized by Eurofins MWG Operon. A single PCR protocol was used to test all primers on every sample using a Biometra “TProfessional” thermal cycler. The PCR for a 10 μL reaction was as follows: 10 ng DNA, dNTPs at 0.2 mM, each primer at 2.5 ng/μL, MgCl_2_ at 1.5 mM and 0.25 units of GoTaq® DNA Polymerase from Promega (Madison, USA) and the parameters were: 94°C for 2 min; 30 cycles of : 94°C for 1 min, 58-60°C for 1 min, 72°C for 1 min; 72°C for 10 min. The PCR products were stained with ethidium bromide and viewed over UV light after agarose gel electrophoresis.

### Cleaning and sequencing

The desired amplicons were cleaned using the JET quick PCR product purification kit (Genomed), then resuspended in water. Cloning of several genes was required prior to sequencing (see below). Cycle sequencing reactions used the BigDye® Terminator v3.1 kit and the manufacturer’s protocols. The sequencing primers were the same as used for the PCR. Cycle sequencing products were cleaned following the ethanol precipitation protocol of Applied Biosystems. The samples were sequenced with an Applied Biosystems 3130xl Genetic Analyzer.

### Cloning

Cloning was performed to resolve the unique sequences of alleles and orthologs for several of the genes including *4CL2*, *CAD*, *CesA1*, *GA*-*20*, *Knat7*, *Kor1*, *Mybr2r3* and *SAD*. Chemically competent XLI-blue strain *Escherichia coli* cells were prepared using a modified protocol from Inoue et al. [49]. PCR products were then inserted into plasmids using the CloneJET PCR cloning kit from Thermo Scientific and following the manufacturer’s protocol. The transformed bacteria were left to grow overnight at 37°C on Luria-Bertani (LB) plates containing 50 μg/mL ampicillin. Eight colonies were then resuspended into LB and 1.5 μL was used in a standard 15 μL PCR mix using the same primers as the initial PCR amplification. Electrophoresis gels were used to confirm the presence of the insert. At least 4 colonies per transformation were then sequenced.

### Data analyses

Sequences were analyzed for SNPs and insertions-deletions (indels), using Seqscape Version 2.6, software (Applied Biosystems). This software aligns and reports variations between the different samples. BLAST searches were made against the nucleotide database of the NCBI [50,51] and against Phytozome (http://www.phytozome.net/poplar) to check similarity with existing database sequences. Alignments were made against *Populus trichocarpa* Phytozome gene models but were also compared with the best BLAST hits from other poplar accessions (Table 2). Phylogenetic reconstruction of the genes used in this study has been performed to test for possible paralogy. A gene family keyword search was performed into phytozome (http://www.phytozome.net/search.php) for each gene and *A*. *thaliana* and *P*. *trichocarpa* genes were retrieved and aligned to a consensus *Salix* sequence generated from our study. Midpoint rooted maximum likelihood trees were generated in PhyML (http://www.atgc-montpellier.fr/phyml/) with the HKY85+G substitution model (estimated gamma shape parameter and four substitution rate categories) and NNI branch swapping. Bootstrap values were generated using with 1000 replicates of the same model with NNI swapping.

Variation among *Sali*x species was assessed by calculating: (SNPs/length) × 1,000 over the consensus sequences obtained from the Seqscape alignments. *Populus* homologs were downloaded from GenBank and included in the analysis to test for variation between *Sali*x and *Populus* and to assess inter species variation in the sister genera. The reading frame was determined using DnaSP version 5.10 [52]. Inference of haplotypes was carried out using Arlequin 3.5 [53]. Haplotype number in sequences obtained from the cloning step was also calculated in Arlequin 3.5.

Phylogenetic trees were generated in PAUP [54] with a maximum parsimony analysis or PhyML (http://www.atgc-montpellier.fr/phyml/) with maximum likelihood. Parsimony analyses used 1000 random addition sequences with TBR branch swapping. Parsimony bootstrapping was conducted with 1000 replicates and NNI branch swapping. Maximum likelihood analyses in PhyML were generated with the HKY85+G substitution model (as above) for the orthology testing. All trees were midpoint rooted.

Regression analysis in Excel was used to explore the relationship between DNA ploidy and number of SNPs/kbp on 20 *Salix* genotypes.

## Abbreviations

BLAST: Basic local alignments search tool; EST: Expressed sequence tag; NCBI: National Center for Biotechnology Information; PCR: Polymerase chain reaction; SNP: Single nucleotide polymorphism; SRC: Short rotation coppice.

## Competing interests

The authors declare that they have no competing interests.

## Authors’ contributions

AP performed the experiments, analysed the data and wrote the paper. CK contributed to experimental design, analysis and writing of the manuscript. TH contributed to experimental design, analysis and writing of the manuscript. GD contributed to experimental design and writing of the manuscript. All authors read and approved the final manuscript.

## Supplementary Material

Additional file 1 Details of the amplification results obtained from the loci and species studied from electrophoresis gels for the 27 gene fragments (0: nothing has been amplified; 1, 2, 3, X: 1, 2, 3, multiple band(s)). Click here for file

Additional file 2 **Details of SNPs numbers per studied species per gene region compared to the Phytozome gene model used as reference (see Table **1**).** Click here for file

## References

[B1] Argus GW Infrageneric classification of *Salix* (Salicaceae) in the New World Syst Bot Monogr 1997 52 1 121

[B2] Newsholme C Willows: the genus Salix 1992 Illustrated London, UK: BT Batsford Ltd

[B3] Styles D Thorne F Jones MB Energy crops in Ireland: An economic comparison of willow and Miscanthus production with conventional farming systems Biomass Bioenergy 2008 32 407 421 10.1016/j.biombioe.2007.10.012

[B4] Styles D Jones MB Energy crops in Ireland: quantifying the potential life-cycle greenhouse gas reductions of energy-crop electricity Biomass Bioenergy 2007 31 759 772 10.1016/j.biombioe.2007.05.003

[B5] McLaughlin SB Samson R Bransby D Wiselogel A Evaluating physical, chemical, and energetic properties of perennial grasses as biofuels Bioenergy ’96: Partnerships to Develop and Apply Biomass Technologies Proceedings of the Seventh National Bioenergy Conference; Nashville, TN, USA 1996 1 823936233

[B6] Davin LB Lewis NG Stafford HA, Ibrahim RK Phenylpropanoid metabolism: Biosynthesis of monolignols, lignans and neolignans, lignins and suberins Phenolic Metabolism in Plants 1992 New York: Plenum Press 325 375

[B7] Douglas C Phenylpropanoid metabolism and lignin biosynthesis: from weeds to trees Trends Plant Sci 1996 1 171 178 10.1016/1360-1385(96)10019-4

[B8] Novaes E Kirst M Chiang V Winter-Sederoff H Sederoff R Lignin and Biomass: a negative correlation for wood formation and lignin content in trees Plant Physiol 2010 154 555 561 10.1104/pp.110.161281 20921184 PMC2949025

[B9] Humphreys JM Chapple C Rewriting the lignin roadmap Curr Opin Plant Biol 2002 5 224 229 10.1016/S1369-5266(02)00257-1 11960740

[B10] Taylor NG Cellulose biosynthesis and deposition in higher plants New Phytol 2008 178 239 252 10.1111/j.1469-8137.2008.02385.x 18298430

[B11] Joshi CP Mansfield SD The cellulose paradox - simple molecule, complex biosynthesis Curr Opin Plant Biol 2007 10 220 226 10.1016/j.pbi.2007.04.013 17468038

[B12] Tuskan GA DiFazio S Jansson S Bohlmann J Grigoriev I Hellsten U Putnam N Ralph S Rombauts S Salamov A The genome of black cottonwood, *Populus trichocarpa* (Torr. & Gray) Science 2006 313 1596 1604 10.1126/science.1128691 16973872

[B13] Kelleher CT Chiu R Shin H Bosdet IE Krzywinski MI Fjell CD Wilkin J Yin TM DiFazio SP Ali J A physical map of the highly heterozygous *Populus* genome: integration with the genome sequence and genetic map and analysis of haplotype variation Plant Journal 2007 50 1063 1078 10.1111/j.1365-313X.2007.03112.x 17488239

[B14] Yin T DiFazio SP Gunter LE Zhang X Sewell MM Woolbright SA Allan GJ Kelleher CT Douglas CJ Wang M Tuskan GA Genome structure and emerging evidence of an incipient sex chromosome in *Populus* Genome Res 2008 18 422 430 10.1101/gr.7076308 18256239 PMC2259106

[B15] Geisler-Lee J Geisler M Coutinho PM Segerman B Nishikubo N Takahashi J Aspeborg H Djerbi S Master E Andersson-Gunneras S Poplar carbohydrate-active enzymes. Gene identification and expression analyses Plant Physiol 2006 140 946 962 10.1104/pp.105.072652 16415215 PMC1400564

[B16] Jorge V Dowkiw A Faivre-Rampant P Bastien C Genetic architecture of qualitative and quantitative *Melampsora larici*-*populina* leaf rust resistance in hybrid poplar: genetic mapping and QTL detection New Phytol 2005 167 113 127 10.1111/j.1469-8137.2005.01424.x 15948835

[B17] Kelleher CT Wilkin J Zhuang J Javier Cortes A Pérez Quintero AL Gallagher T Bohlmann J Douglas C Ellis BE Ritland K SNP discovery, gene diversity and linkage disequilibrium in wild populations of *Populus tremuloides* Tree Genet Genomes 2012 8 821 829 10.1007/s11295-012-0467-x

[B18] Berlin S Lagercrantz U von Arnold S Ost T Ronnberg-Wastljung AC High-density linkage mapping and evolution of paralogs and orthologs in *Salix* and *Populus* BMC Genomics 2010 11 20178595 10.1186/1471-2164-11-129 PMC2834636

[B19] Hanley SJ Mallott MD Karp A Alignment of a *Salix* linkage map to the *Populus* genomic sequence reveals macrosynteny between willow and poplar genomes Tree Genet Genomes 2006 3 35 48 10.1007/s11295-006-0049-x

[B20] Cukovic D Ehlting J VanZiffle JA Douglas CJ Structure and evolution of 4-coumarate : coenzyme A ligase (4CL) gene families Biol Chem 2001 382 645 654 11405227 10.1515/BC.2001.076

[B21] Djerbi S Lindskog M Arvestad L Sterky F Teeri TT The genome sequence of black cottonwood (*Populus trichocarpa*) reveals 18 conserved cellulose synthase (*CesA*) genes Planta 2005 221 739 746 10.1007/s00425-005-1498-4 15940463

[B22] Meyermans H Morreel K Lapierre C Pollet B De Bruyn A Busson R Herdewijn P Devreese B Van Beeumen J Marita JM Modifications in lignin and accumulation of phenolic glucosides in poplar xylem upon down-regulation of caffeoyl-coenzyme A O-methyltransferase, an enzyme involved in lignin biosynthesis J Biol Chem 2000 275 36899 36909 10.1074/jbc.M006915200 10934215

[B23] Barker JHA Pahlich A Trybush S Edwards KJ Karp A Microsatellite markers for diverse Salix species Mol Ecol Notes 2003 3 4 6

[B24] Przyborowski JA Sulima P The analysis of genetic diversity of *Salix viminalis* genotypes as a potential source of biomass by RAPD markers Ind Crops Prod 2010 31 395 400 10.1016/j.indcrop.2009.12.009

[B25] Barker JHA Matthes M Arnold GM Edwards KJ Ahman I Larsson S Karp A Characterisation of genetic diversity in potential biomass willows (Salix spp.) by RAPD and AFLP analyses Genome 1999 42 173 183 10231956

[B26] Thibault J Nuclear DNA amount in pure species and hybrid willows (*Salix*): a flow cytometric investigation Can J Bot 1998 76 157 165

[B27] Meikle RD Willows and poplars of Great Britain and Ireland. illustrated, reprint edn 1984 Botanical Society of the British Isles: London, UK

[B28] Loureiro J Rodriguez E Dolezel J Santos C Two new nuclear isolation buffers for plant DNA flow cytometry: a test with 37 species Ann Bot 2007 100 875 888 10.1093/aob/mcm152 17684025 PMC2749623

[B29] Guindon S Dufayard J-F Lefort V Anisimova M Hordijk W Gascuel O New algorithms and methods to estimate maximum-likelihood phylogenies: assessing the performance of PhyML 3.0 Syst Biol 2010 59 307 321 10.1093/sysbio/syq010 20525638

[B30] Stamati K Blackie S Brown JWS Russell J A set of polymorphic SSR loci for subarctic willow (*Salix lanata*, *S*-*lapponum* and *S*-*herbacea*) Mol Ecol Notes 2003 3 280 282 10.1046/j.1471-8286.2003.00426.x

[B31] MacAlpine WJ Shield IF Trybush SO Hayes CM Karp A Overcoming barriers to crossing in willow (Salix spp.) breeding Aspects of Appl Biol 2008 90 173 180

[B32] Berlin S Fogelqvist J Lascoux M Lagercrantz U Ronnberg-Wastljung AC Polymorphism and divergence in two willow species, *Salix viminalis* L. and *Salix schwerinii* E. Wolf G3 (Bethesda, Md) 2011 1 387 400 2011 10.1534/g3.111.000539 PMC3276148 22384349

[B33] Hanley SJ Pei MH Powers SJ Ruiz C Mallott MD Barker JHA Karp A Genetic mapping of rust resistance loci in biomass willow Tree Genet Genomes 2011 7 597 608 10.1007/s11295-010-0359-x

[B34] Azuma T Kajita T Yokoyama J Ohashi H Phylogenetic relationships of *Salix* (Salicaceae) based on *rbcL* sequence data Am J Bot 2000 87 67 75 10.2307/2656686 10636831

[B35] Hardig TM Anttila CK Brunsfeld SJ A phylogenetic analysis of salix (Salicaceae) based on matk and ribosomal DNA sequence data J Bot 2010 2010

[B36] Trybush S Jahodova S Macalpine W Karp A A genetic study of a *Salix* germplasm resource reveals new insights into relationships among subgenera, sections and species Bioenergy Res 2008 1 67 79 10.1007/s12155-008-9007-9

[B37] Brunsfeld SJ Soltis DE Soltis PS Evolutionary patterns and processes in *Salix* sect. *Longifoliae*: evidence from chloroplast DNA Syst Bot 1992 17 239 256 10.2307/2419520

[B38] Ingvarsson PK Nucleotide polymorphism and linkage disequilbrium within and among natural populations of European Aspen (*Populus tremula* L., Salicaceae) Genetics 2005 169 945 953 10.1534/genetics.104.034959 15489521 PMC1449109

[B39] Ingvarsson PK Multilocus patterns of nucleotide polymorphism and the demographic history of *Populus tremula* Genetics 2008 180 329 340 10.1534/genetics.108.090431 18716330 PMC2535685

[B40] Pei MH Lindegaard K Ruiz C Bayon C Rust resistance of some varieties and recently bred genotypes of biomass willows Biomass & Bioenergy 2008 32 453 459 10.1016/j.biombioe.2007.11.00223936633

[B41] Suda Y Argus GW Chromosome numbers of some North American *Salix* Brittonia 1968 20 191 197 10.2307/2805440

[B42] Hamberger B Ellis M Friedmann M de Azevedo Souza C Barbazuk B Douglas CJ Genome-wide analyses of phenylpropanoid-related genes in Populus trichocarpa, Arabidopsis thaliana, and Oryza sativa: the Populus lignin toolbox and conservation and diversification of angiosperm gene families Can J Bot 2007 85 1182 1201 10.1139/B07-098

[B43] Weng J-K Chapple C The origin and evolution of lignin biosynthesis New Phytol 2010 187 273 285 10.1111/j.1469-8137.2010.03327.x 20642725

[B44] Li L Cheng XF Leshkevich J Umezawa T Harding SA Chiang VL The last step of syringyl monolignol biosynthesis in angiosperms is regulated by a novel gene encoding sinapyl alcohol dehydrogenase Plant Cell 2001 13 1567 1586 11449052 10.1105/TPC.010111 PMC139549

[B45] Ramsay H Rieseberg LH Ritland K The correlation of evolutionary rate with pathway position in plant terpenoid biosynthesis Mol Biol Evol 2009 26 1045 1053 10.1093/molbev/msp021 19188263

[B46] Barakate A Stephens J Goldie A Hunter WN Marshall D Hancock RD Lapierre C Morreel K Boerjan W Halpin C Syringyl lignin is unaltered by severe sinapyl alcohol dehydrogenase suppression in tobacco The Plant Cell Online 2011 23 4492 4506 10.1105/tpc.111.089037 PMC3269879 22158465

[B47] Bomati EK Noel JP Structural and kinetic basis for substrate selectivity in *Populus tremuloides* sinapyl alcohol dehydrogenase Plant Cell 2005 17 1598 1611 10.1105/tpc.104.029983 15829607 PMC1091777

[B48] Eun HM Enzymology Primer for Recombinant DNA Technology 1996 San Diego: Academic press

[B49] Inoue H Nojima H Okayama H High-efficiency transformation of *Escherichia*-*coli* with plasmids Gene 1990 96 23 28 10.1016/0378-1119(90)90336-P 2265755

[B50] Altschul SF Gish W Miller W Myers EW Lipman DJ Basic Local Alignment Search Tool J Mol Biol 1990 215 403 410 2231712 10.1016/S0022-2836(05)80360-2

[B51] Johnson M Zaretskaya I Raytselis Y Merezhuk Y McGinnis S Madden TL NCBI BLAST: a better web interface Nucleic Acids Res 2008 36 W5 W9 10.1093/nar/gkn201 18440982 PMC2447716

[B52] Librado P Rozas J DnaSP v5: a software for comprehensive analysis of DNA polymorphism data Bioinformatics 2009 25 1451 1452 10.1093/bioinformatics/btp187 19346325

[B53] Excoffier L Laval G Schneider S Arlequin (version 3.0): An integrated software package for population genetics data analysis Evol Bioinf 2005 1 47 50 PMC2658868 19325852

[B54] Swofford D PAUP*. Phylogenetic Analysis Using Parsimony (*and other methods). Version 4. Sinauer Associates 2002

