# Supplementary material for: High levels of variation in Salix lignocellulose genes revealed using poplar genomic resources
Source: Biotechnol Biofuels. 2013 Aug 7;6:114. doi: 10.1186/1754-6834-6-114 (PMC3750764; doi:10.1186/1754-6834-6-114)
Supplement: Additional file 1 — Details of the amplification results obtained from the loci and species studied from electrophoresis gels for the 27 gene fragments (0: nothing has been amplified; 1, 2, 3, X: 1, 2, 3, multiple band(s)). [file 1754-6834-6-114-S1.docx]

Additional file 1 Details of the amplification results obtained from the loci and species studied from electrophoresis gels for the 27 gene fragments (0: nothing has been amplified; 1, 2, 3, X: 1, 2, 3, multiple band(s))

|  | 4CL2 | C3H1 | C4H | CesA2 | HCT | PAL1 | CesA1 | CAD | SAD | GA-20 | MYBR2R3 | *Knat7* | Kor1 | BZIP9 | 4CL1prom | 4CL1 | CCoAOMT | CCR | MYB | MYB63 | PTOMT1 | Sucrose | PAL2 | F5H | AP2 | BHLH144 | BZIP47 |
| --- | --- | --- | --- | --- | --- | --- | --- | --- | --- | --- | --- | --- | --- | --- | --- | --- | --- | --- | --- | --- | --- | --- | --- | --- | --- | --- | --- |
| *Populus tremula* L. | 1 | 1 | 1 | 1 | 1 | 1 | 1 | 2 | 2 | 3 | 2 | 2 | 1 | 1 | 3 | 2 | 1 | 1 | 2 | 1 | 1 | 2 | X | 1 | 1 | 1 | X |
| *S. caprea* L. | 1 | 1 | 0 | 1 | 0 | 1 | 0 | 2 | 1 | 0 | 1 | 1 | 1 | 0 | 0 | 0 | 0 | 0 | 0 | 0 | 0 | 0 | 1 | 0 | 0 | 0 | X |
| *S. viminalis* L. | 1 | 1 | 1 | 1 | 0 | 1 | 2 | 2 | X | 1 | 2 | 2 | 1 | 0 | 0 | 0 | 0 | 0 | 0 | 0 | 0 | 0 | X | 2 | 1 | 0 | X |
| *S. cinerea* 'Tricolor' | 1 | 1 | 0 | 1 | 0 | 1 | 0 | 2 | 1 | 1 | 2 | 2 | 2 | 0 | 0 | 0 | 0 | 0 | 0 | 0 | 0 | 0 | 2 | 0 | 0 | 0 | X |
| *S. babylonica var. pekinensis* 'Tortuosa' | 1 | 1 | 1 | 1 | 1 | 1 | 1 | 2 | 1 | 1 | 2 | 2 | 2 | 0 | 0 | 0 | 1 | 0 | 0 | 0 | 0 | 0 | 2 | 1 | 1 | 0 | X |
| *S. aurita* L. | 1 | 1 | 1 | 1 | 0 | 1 | 2 | 2 | 1 | 1 | 2 | 2 | 1 | 0 | 0 | 0 | 0 | 1 | 0 | 0 | 0 | 0 | 3 | 1 | 2 | 0 | X |
| *S. herbacea* L. | 1 | 1 | 1 | 1 | 0 | 1 | 2 | 2 | 1 | 1 | 0 | 2 | 2 | 0 | 0 | 0 | 0 | 0 | 0 | 0 | 0 | 0 | 2 | 0 | 0 | 0 | X |
| *S. alba* L. *var. vitellina* L. Stokes | 1 | 1 | 1 | 1 | 0 | 1 | 1 | 2 | 1 | 1 | 2 | 2 | 2 | 1 | 0 | 0 | 1 | 0 | 0 | 0 | 0 | 0 | 2 | 1 | 0 | 1 | X |
| *S. gracilistyla* Miq. *var. melanostachys* | 1 | 1 | 1 | 1 | 1 | 1 | 2 | 2 | 2 | 1 | 2 | 1 | 2 | 0 | 0 | 0 | 0 | 0 | 0 | 0 | 0 | 0 | 1 | 0 | 1 | 0 | X |
| *S. glabra* Scop. | 1 | 1 | 1 | 1 | 1 | 1 | 2 | 2 | X | 1 | 2 | 2 | 2 | 0 | 0 | 0 | 0 | 1 | 0 | 0 | 0 | 0 | 2 | X | 2 | 2 | X |
| *S. phylicifolia* L. | 1 | 1 | 1 | 1 | 1 | 1 | 2 | 2 | X | 1 | 2 | 2 | 2 | 0 | 0 | 0 | 0 | 0 | 0 | 0 | 0 | 0 | 2 | 1 | 2 | 2 | X |
| *S. lucida* Muhlenb. | 1 | 1 | 0 | 1 | 1 | 1 | 2 | 1 | 2 | 1 | 2 | 2 | 2 | 0 | 0 | 0 | 0 | 0 | 0 | 0 | 0 | 0 | 2 | 2 | 2 | 0 | X |
| *S. rehderiana* Schneid. | 1 | 1 | 0 | 1 | 0 | 1 | 1 | 2 | 1 | 1 | 2 | 2 | 2 | 0 | 0 | 0 | 0 | 0 | 0 | 0 | 0 | 0 | 2 | 1 | 0 | 1 | X |
| *S. exigua* L. | 1 | 1 | 1 | 1 | 1 | 1 | 2 | 2 | 2 | 1 | 2 | 2 | 2 | 0 | 0 | 0 | 0 | 1 | 0 | 0 | 0 | 0 | 2 | 1 | 0 | 1 | X |
| *S. pentandra* L. | 1 | 1 | 1 | 1 | 1 | 1 | 2 | 2 | 1 | 1 | 2 | 2 | 2 | 0 | 0 | 0 | 1 | 0 | 0 | 0 | 0 | 0 | 2 | 2 | 1 | 2 | X |
| *S. elaeagnos* Scop. | 1 | 1 | 1 | 1 | 1 | 1 | 2 | 2 | 1 | 1 | 1 | 2 | 1 | 0 | 0 | 0 | 0 | 1 | 0 | 0 | 0 | 0 | 1 | X | 2 | 0 | X |
| *S. fragilis* L. | 1 | 1 | 1 | 1 | 0 | 1 | 1 | 0 | 1 | 1 | 2 | 2 | 2 | 0 | 0 | 0 | 0 | 0 | 0 | 0 | 0 | 0 | 2 | 0 | 1 | 0 | X |
| *S. phylicifolia* L. | 1 | 1 | 0 | 1 | 0 | 1 | 0 | 2 | 3 | 1 | 1 | 0 | 1 | 0 | 0 | 0 | 0 | 0 | 0 | 0 | 0 | 0 | 1 | 0 | 0 | 0 | 0 |
| *S. scouleriana* Barr. | 1 | 1 | 0 | 1 | 0 | 1 | 2 | 1 | 2 | 1 | 1 | 2 | 1 | 0 | 0 | 0 | 0 | 0 | 0 | 0 | 0 | 0 | 1 | 0 | 0 | 0 | 0 |
| *S. purpurea* L. | 1 | 1 | 1 | 1 | 1 | 1 | 2 | 2 | 1 | 1 | 1 | 2 | 2 | 0 | 0 | 0 | 0 | 1 | 0 | 0 | 0 | 0 | X | X | 2 | X | X |
| *S. viminalis* L. | 1 | 1 | 1 | 0 | 1 | 1 | 2 | 2 | X | 1 | 2 | 2 | 1 | 0 | 0 | 0 | 0 | 0 | 0 | 0 | 0 | 0 | X | X | 1 | X | X |
| *S. viminalis* L. | 1 | 1 | 1 | 1 | 0 | 1 | 2 | 1 | 1 | 1 | 2 | 2 | 2 | 0 | 0 | 0 | 0 | 1 | 0 | 0 | 0 | 0 | X | X | 1 | X | X |
| *S. purpurea* L. | 1 | 1 | 1 | 1 | 0 | 1 | 2 | 1 | 1 | 1 | 2 | 2 | 1 | 0 | 0 | 0 | 0 | 0 | 0 | 0 | 0 | 0 | X | X | 2 | X | X |
| Sven | 1 | 1 | 1 | 1 | 0 | 1 | 2 | 2 | X | 1 | 2 | 2 | 2 | 0 | 0 | 0 | 0 | 0 | 0 | 0 | 0 | 0 | X | 2 | 0 | 0 | X |
| Inger | 1 | 1 | 1 | 1 | 0 | 1 | 2 | 2 | X | 1 | 2 | 0 | 2 | 0 | 0 | 0 | 0 | 1 | 0 | 0 | 0 | 0 | X | 2 | 0 | 0 | X |
| Tordis | 1 | 1 | 1 | 1 | 0 | 1 | 2 | 2 | 1 | 2 | 2 | 2 | 2 | 0 | 0 | 0 | 0 | 1 | 0 | 0 | 0 | 0 | 1 | 2 | 0 | 0 | X |
| Endurance | 1 | 1 | 1 | 1 | 0 | 1 | 1 | 2 | X | 1 | 2 | 2 | 2 | 0 | 0 | 0 | 0 | 0 | 0 | 0 | 0 | 0 | X | 0 | 0 | 0 | X |
| Tora | 1 | 1 | 0 | 1 | 1 | 1 | 2 | 2 | X | 2 | 2 | 2 | 2 | 0 | 0 | 0 | 0 | 0 | 0 | 0 | 0 | 0 | X | 2 | 0 | 0 | X |
| Resolution | 1 | 1 | 1 | 1 | 1 | 1 | 2 | 2 | X | 2 | 2 | 2 | 2 | 0 | 0 | 0 | 0 | 1 | 0 | 0 | 0 | 0 | X | 2 | 1 | 0 | 1 |
| Doris | 1 | 1 | 1 | 1 | 1 | 1 | 2 | 2 | X | 2 | 2 | 2 | 2 | 0 | 0 | 0 | 0 | 1 | 0 | 0 | 0 | 0 | X | 2 | 1 | 1 | X |
| Terra Nova | 1 | 1 | 1 | 1 | 1 | 1 | 2 | 2 | X | 2 | 2 | 2 | 2 | 0 | 0 | 0 | 0 | 0 | 0 | 0 | 0 | 0 | 1 | 1 | 2 | 0 | X |
| Torhild | 1 | 1 | 1 | 1 | 1 | 1 | 2 | 2 | 2 | 2 | 2 | 2 | 1 | 0 | 0 | 0 | 0 | 0 | 0 | 0 | 0 | 0 | X | 2 | 1 | 0 | 1 |
| *S.* 𝗑 *smithiana* Willd | 1 | 1 | 1 | 1 | 1 | 1 | 2 | 2 | X | 1 | 2 | 2 | 1 | 0 | 0 | 0 | 0 | 0 | 0 | 0 | 0 | 0 | X | X | 1 | 0 | X |
| *S.* 𝗑 *rubens* 'Basfordiana' | 1 | 1 | 1 | 0 | 0 | 1 | 0 | 2 | 1 | 3 | 2 | 2 | 2 | 1 | 0 | 0 | 1 | 0 | 0 | 0 | 0 | 0 | 2 | 1 | 0 | 0 | X |
| *S.* 𝗑 *laurina* Sm. | 1 | 1 | 1 | 1 | 0 | 1 | 1 | 2 | 1 | 1 | 2 | 2 | 2 | 0 | 0 | 0 | 0 | 0 | 0 | 0 | 0 | 0 | 2 | 1 | 2 | 0 | X |
| *S.* 𝗑 *erdingeri* Kern | 1 | 1 | 1 | 1 | 1 | 1 | 2 | 2 | X | 1 | 2 | 2 | 2 | 0 | 0 | 0 | 0 | 1 | 0 | 0 | 0 | 0 | 2 | 0 | 2 | 1 | X |
| *S.* 𝗑 *rubens* Schrank | 1 | 1 | 1 | 1 | 0 | 1 | 1 | 2 | 1 | 1 | 2 | 2 | 2 | 1 | 0 | 0 | 1 | 0 | 0 | 0 | 0 | 0 | 2 | 0 | 1 | 2 | X |
| *S.* 𝗑 *rubra* Huds. | 1 | 1 | 1 | 1 | 1 | 1 | 2 | 2 | 2 | 1 | 2 | 2 | 2 | 0 | 0 | 0 | 0 | 1 | 0 | 0 | 0 | 0 | 2 | 1 | 2 | 0 | X |
| *S.* 𝗑 *chrysocoma* Dode | 1 | 1 | 1 | 1 | 1 | 1 | 2 | 2 | 2 | 1 | 2 | 2 | 2 | 0 | 0 | 0 | 1 | 0 | 0 | 0 | 0 | 0 | 2 | 2 | 1 | 2 | X |
| *S.* 𝗑 *erythroflexuosa* Rag. | 1 | 1 | 1 | 1 | 1 | 1 | 2 | 2 | 1 | 1 | 2 | 2 | 2 | 0 | 0 | 0 | 1 | 0 | 0 | 0 | 0 | 0 | 2 | 1 | 0 | 1 | X |
| *S.* 𝗑 *rubens* Schrank *var. sanguinea* 'Basfordiana' | 1 | 1 | 1 | 1 | 0 | 1 | 2 | 1 | 1 | 1 | 2 | 2 | 2 | 1 | 0 | 0 | 1 | 0 | 0 | 0 | 0 | 0 | X | 1 | 0 | 2 | X |
