# Supplementary material for: High levels of variation in Salix lignocellulose genes revealed using poplar genomic resources
Source: Biotechnol Biofuels. 2013 Aug 7;6:114. doi: 10.1186/1754-6834-6-114 (PMC3750764; doi:10.1186/1754-6834-6-114)
Supplement: Additional file 2 — Details of SNPs numbers per studied species per gene region compared to the Phytozome gene model used as reference (see Table 1). [file 1754-6834-6-114-S2.docx]

Additional file 2 Details of SNPs numbers per studied species per gene region compared to the Phytozome gene model used as reference (see Table 1)

| **Commercial clones** |  | C3H1 | C4H | CesA1 | CesA2 | Kor1 | Pal1 | SAD | *Knat7* |
| --- | --- | --- | --- | --- | --- | --- | --- | --- | --- |
| ‘Sven’ | *S. viminalis* x (*S. schwerinii* x *S. viminalis*) | 24 | 35 |  | 22 | 15 | 20 | - | 11 |
| ‘Inger’ | *S. triandra* x *S. viminalis* | 25 | 35 |  | 24 | 15 | 24 | - | - |
| ‘Tordis’ | (*S. schwerinii* x *S. viminalis*) x *S. viminalis* | 26 | 32 | 42 | 22 | 16 | 20 | - | 12 |
| ‘Endurance’ |  | - | - | - | 25 | - | - | - | - |
| ‘Tora’ | *S. schwerinii* x *S. viminalis* | 26 |  | 41 | 24 | 16 | 20 | - | 13 |
| ‘Resolution’ | (*S. viminalis* x (*S. schwerinii* x *S. viminalis*)) x (*S. viminalis* x (*S. viminalis* x *S. schwerinii*)) | 26 | 34 | 27 | 23 | 15 | 21 | - | 11 |
| ‘Doris’ |  | 24 | 33 | 26 | 24 | 15 | 20 | - | 12 |
| ‘Terra Nova’ | (*S. triandra* x *S. viminalis*) x *S. linderstipularis* | 26 | 35 |  | - | 15 | 20 | - | 13 |
| ‘Torhild’ | (*S. schwerinii* x *S. viminalis*) x *S. viminalis* | 26 | - | 41 | 24 | 15 | 20 | - | 11 |
|  |  |  |  |  |  |  |  |  |  |
| **Hybrids** |  | C3H1 | C4H | CesA1 | CesA2 | Kor1 | Pal1 | SAD | *Knat7* |
| *S.* x *smithiana* Willd. | *S. cinerea* L x *S. viminalis* L. | 25 | 34 | 26 | 24 | 15 | 21 | - | - |
| *S.* x *rubens* 'Basfordiana' | *S. alba* L. var. vitellina L. Stokes x *S. fragilis* L. | 19 |  | - |  | 16 | 17 | 39 | - |
| *S.* x *laurina* Sm. | *S. caprea* L. x *S. phylicipholia* | 24 |  | 23 | 25 | 14 | 17 | 34 | 12 |
| *S.* x *erdingeri* Kern | *S. daphnoides* Vill. x *S. caprea* L. | 23 | 33 | 26 | 24 | 15 |  | - | 14 |
| *S.* x *rubens* Schrank | *S. alba* L. x *S. fragilis* L. | 20 |  | 24 | 25 | 16 | 16 | 37 | - |
| *S.* x *rubra* Huds | (*S. purpurea* x *S. viminalis* L.) | - | - | 25 | - | - | - | - | - |
| *S.* x *chrysocoma* Dode | *S. alba* L. var. vitellina L. Stokes x *S. babylonica* L. | - | - | 24 | - | - | - | 39 | - |
| *S.* x *erythroflexuosa* Rag. | *S. x chrysocoma* x *S. matsudana* | - | - | - | - | - | - | 37 | - |
|  |  |  |  |  |  |  |  |  |  |
| **Species** |  | C3H1 | C4H | CesA1 | CesA2 | Kor1 | Pal1 | SAD | *Knat7* |
| *S. caprea* L. |  | 24 |  | - | 23 | 15 | 18 | 36 | 18 |
| *S. viminalis* L. |  | 25 | 34 | 26 | 24 | 15 | 21 | - | 11 |
| *S. cinerea* 'Tricolor' |  | 24 |  | - | 25 | 14 | 19 | 36 | 12 |
| *S. babylonica* var. pekinensis 'Tortuosa' |  | 20 |  | 25 | 24 | 16 |  | 36 | - |
| *S. aurita* L. |  | 23 |  | 23 | 25 | 17 | 21 | 33 | 12 |
| *S. herbacea* |  | 26 | 31 | 26 | 21 | 18 | 19 | 39 | 14 |
| *S. alba* L. var. vitellina L. Stokes |  | 20 | 31 | 24 | 22 | 17 |  | 40 | - |
| *S. gracilistyla* Miq. var. melanostachys |  | 23 | 35 | 35 | 23 | 17 | 20 | 35 | 11 |
| *S. glabra* Scop. |  | 22 | 32 | 24 | 24 | 14 | 18 | - | 11 |
| *S. phylicifolia* L. |  | 23 |  | 26 | 23 | 14 | 19 | - | 12 |
| *S. lucida* Muhlenb. |  | - | - | 26 | - | - | - | - | - |
| *S. rehderiana* Schneid. |  | - | - | - | - | - | - | 35 | - |
| *S. pentandra* L. |  | - | - | - | - | - | - | 36 | - |
| *S. fragilis* L. |  | - | - | - | - | - | - | 35 | - |
